# Supplementary material for: Practical research on the boundaries of MAYA design principles with ceramic products as the carrier
Source: PLoS One. 2026 Apr 2;21(4):e0342855. doi: 10.1371/journal.pone.0342855 (PMC13046151; doi:10.1371/journal.pone.0342855)
Supplement: S1 Checklist — (PDF) [file pone.0342855.s003.pdf]

Inclusivity in global research

PLOS' policy on inclusivity in global research aims to improve transparency in the reporting of research performed outside of researchers' own country or community and ensures that PLOS publications reporting global research adhere to high standards for research ethics and authorship. Authors of relevant research articles may be asked to complete the questionnaire below, which outlines ethical, cultural, and scientific considerations specific to inclusivity in global research. This questionnaire may be requested when researchers have travelled to a different country to conduct research, if research uses samples collected in another country, research with Indigenous populations or their lands, or if research is on cultural artefacts. Researchers travelling to another country solely to use laboratory equipment will not normally be required to complete the questionnaire. However, the questionnaire can be requested at the journal's discretion for any submission – if you have been requested to complete this questionnaire by the PLOS journal you submitted to, please do so.

Please complete the questionnaire below and include this as a Supporting Information file with your manuscript. Note that if your paper is accepted for publication, this checklist will be published with your article in the supporting information files. Please ensure that you reference the checklist in the main body of your manuscript. We suggest adding a subsection 'Inclusivity in global research' to your Methods section and adding the following sentence: "Additional information regarding the ethical, cultural, and scientific considerations specific to inclusivity in global research is included in the Supporting Information (SX Checklist)"

The questions have been designed to be applicable to a wide range of study types, and there are subsections for both human subjects research and non-human subjects research. If any of the questions are not relevant to your research please mark them as "N/A" as appropriate.

Ethical considerations, permits and authorship

This section is applicable to all research types.

Provide details as to who granted permissions and/or consent for the study to take place in the Methods section of your manuscript. This should include the names of **all** ethics boards, governmental organizations, community leaders or other bodies that provided approval for the study.

Reported on page number: 5

If individuals provided approval refer to these people by their role or title but do not list their name(s).

If there were any deviations from the study protocol after approval was obtained please provide

Reported on page number: 0

details of these changes in the Methods section of your manuscript.

Did this study involve local collaborators that are residents of the country where the research was conducted or members of the community studied? If you do not have any authors from said communities, please provide an explanation for this below.

The study was conducted at Lvliang University, China, with participants consisting of the university's faculty and students. One of the co-authors is a faculty member at Lvliang University and contributed to study design, data collection, and interpretation. The involvement of a local collaborator ensured that the research was carried out in an ethically and culturally appropriate manner within the local academic context.

Everyone listed as an author should meet PLOS' criteria for authorship and all individuals who meet these criteria should be included in the author byline, rather than the acknowledgements. For further information please see the journal's Authorship Policy.

Human subjects research (e.g. health research, medical research, cross-cultural psychology)

Did you obtain written informed consent from a representative of the local community or region before the research took place? How did you establish who speaks for the community? Details of written informed consent obtained from study participants should be reported separately in the Methods section of your manuscript.

Community (institutional) consent:The research was conducted with official approval from Lvliang University, which was considered the representative body of the local academic community. The permission was granted through the university's ethics or research administration prior to data collection.

Participant consent: Each participant received an information sheet describing the study's purpose, procedures, and confidentiality. Written informed consent was obtained from all faculty and student participants prior to participation.

How did members of the local community provide input on the aims of the research investigation, its methodology, and its anticipated outcome(s)?

When engaging with the local community, how did you ensure that the informed consent documents

and other materials could be understood by local stakeholders?

Will the findings of the research be made available in an understandable format to stakeholders in the community where the study was conducted (e.g. via a presentation, summary report, copies of publications, etc.)? Please provide details of how this will be achieved.

Community engagement:As the study was conducted within Lvliang University by a faculty member of the institution, the aims and methodology were developed in consultation with colleagues and administrative staff. Their feedback ensured that the research aligned with the interests and ethical standards of the local academic community.

Informed consent process:The consent form and participant information sheet were prepared in Chinese to ensure clarity. Participants were informed of the study's purpose, procedures, potential risks, and their right to withdraw at any time without consequence. Written informed consent was obtained prior to participation.

Non-human subjects research using specimens/ animals collected as part of the study, or those housed in archival collections. Examples include archaeology, paleontology, botany and zoology.

Did the permission you obtained from a local authority to perform the study include an agreement on access to outputs and benefit sharing? This may include procedures to enable fair distribution of the benefits and resources arising from the research performed. Please include any details of Prior Informed Consent and Benefit Sharing Agreements obtained. These may be required by field-specific regulations, for example the Convention on Biological Diversity (CBD) and the associated Nagoya Protocol.

This study did not involve the collection or analysis of any non-human specimens, animals, or materials housed in archival collections. Therefore, permissions or benefit-sharing agreements under the Convention on Biological Diversity (CBD) were not required.

If the material used in your study was imported, please A) provide the year it was imported and B) indicate whether permits were obtained to import/export the materials used, C) provide details of any permits obtained. If this information is not available, please indicate this.

This study did not involve the import or export of any biological, archaeological, or other physical materials. All data were collected locally at Lvliang University, China. Therefore, no import/export permits were required.

If you used archival specimens, please state how the material used in your study was acquired by the institute it is held in and provide details of any permits obtained for the original excavations/

This study did not use any archival specimens or materials. All data were collected directly from human participants (faculty and students) at Lvliang University, China, through questionnaire surveys. Therefore, no excavation or collection permits were required.

sample collection. If this information is not available, please indicate this.

How was the potential cultural significance of the materials collected in your study to local communities considered in your research design? Were Indigenous peoples and/or local researchers and institutions involved with archaeological excavations / collection of specimens? If so, please

The study did not involve Indigenous communities or archaeological collections. All research activities were conducted within Lvliang University, with the participation of local faculty and students. The inclusion of a local co-author from the university ensured that the research was contextually appropriate and ethically sound.

provide a description of their involvement.

If your manuscript includes photographs of human remains please indicate whether authors

The manuscript does not include photographs of human remains, and the study did not involve any collection or analysis of human remains. Therefore, ethical approval and consent procedures related to human remains are not applicable.

obtained permission from descendants or affiliated cultural communities to do so.
