# Supplementary material for: Practical research on the boundaries of MAYA design principles with ceramic products as the carrier
Source: PLoS One. 2026 Apr 2;21(4):e0342855. doi: 10.1371/journal.pone.0342855 (PMC13046151; doi:10.1371/journal.pone.0342855)
Supplement: Attachment — Submitted filename: Review PLOS ONE Maya Aug 5 2025.docx [file pone.0342855.s004.docx]

Manuscript Number: PONE-D-25-32602

Article Type: Research Article

Full Title: Practical research on the boundaries of MAYA design Principles with Ceramic

Products as the Carrier

REVIEW DATE: Aug 5, 2025

**Overall concept:**

This exquisitely written paper examines the MAYA principle in ceramic products by measuring the effects of the aesthetic properties of typicality and novelty on aesthetic preference. The study focuses on ceramic products, specifically two distinct categories: open and closed. The authors included 10 stimuli for the open category and 10 for the closed, selecting vases and lighting objects to represent both categories. A within-subjects experimental design was implemented to test the objectives using Chinese participants. Measures were taken to avoid order effects on both the stimuli and the measures. The study demonstrates that the MAYA principle applies to both categories and further confirms the moderating role of category type.

I really like the study, even though it includes only one experiment. Below, I have included some suggestions to help make the paper more rigorous and clear for readers. I believe it is an excellent study that deserves publication. Many of my suggestions are cosmetic, but some address justification and clarification. Because this is a study in the social sciences in PLOS ONE—even though it is focused on product design and consumer behavior—I am convinced that the inclusion of hypotheses is necessary. That is the major change I request. So, it is small major revision in my opinion.

In Summary, these are the main changes I believe will improve the manuscript:

- **Clarify writing and improve consistency:** Revise unclear sentences (e.g., in the abstract) and maintain consistent order when presenting key concepts like typicality vs. novelty and vases vs. lighting.
- **Strengthen methodological transparency:** Add details about the expert panel, stimulus selection, and measurement procedures, including justifications for construct-scales choices and sources.
- **Enhance rigor and scientific contribution:** Include formal hypotheses, verify construct definitions and usage (e.g., unity or typicality? Aesthetic pleasure or aesthetic preference?), and support theoretical claims in the discussion with relevant citations.
- **Improve presentation of results and key findings:** Make figures and tables easier to interpret by adding labels, totals, and clarifications; visually highlight key values. Explicitly state in the conclusion that the study confirms the MAYA principle across both product categories.

Details of these changes are provided in the following section-by-section comments:

**Abstract**

This idea is not clear: “Both typicality and novelty shape aesthetic preference—how closely a product aligns with category norms—and perceived originality.” It implies that “how closely a product aligns with category norms” relates to aesthetic preference, and not typicality necessarily. The second part of the phrase is incomplete. Please revise.

In this “21.8\% of the variance in aesthetic preference for ceramic vases and 20.3\%” is that two typos? See back slash before percentage signs.

**Introduction**

As this is not a descriptive study, it is necessary to include hypotheses and link the results and discussion to those proposed hypotheses. Although the objectives are clearly stated, hypothesis testing will make the study more rigorous and scientific.

**Materials and methods**

There is a need to include more detail and measures of the expert panel. How many experts? Criteria for selection? How did they rate the stimuli? How many did you start with to finish with 10 products per category? Where did you get the images from?

**Procedures**

I really like what you did to avoid order effects.

You state “These statements about aesthetic preference, novelty, and typicality were adapted from previously validated research [33,34]”. However, references 33 and 34 only address “Aesthetic Pleasure”, which is similar but different from aesthetic preference. You need to justify why you used that construct of aesthetic pleasure, and not one for aesthetic preference AP--- there are various authors with scales for AP. Besides, do those references 33-34 include measures on typicality and novelty? I do not think so, please revise. To make sure it is clear which measures you used, it would be helpful to include a table with the scales, items, loadings, and Cronback alpha. Or did you use 1-item measures? Please indicate your items exactly and explicitely. It is important to see how you measured the constructs, number of items, and which references did you use.

**Results**

For better understanding Fig. 2 and Fig. 3, I suggest putting the stimuli (tiny image) below each number. It is hard to read the figures when the stimuli is in another page in Fig. 1.

The same goes for Tables 3 and 4. Why not add a column to include the stimuli in the table? Besides, it is not clear what the numbers are. For example, what is “2.24(1.10)”? Meand (SD)? Even if the text explains it, the table should be understandable without reading the text.

In Tables 3 and 4 will be useful to include a total at a new bottom line.

To help readers identify the highest and lowest ratings, you could highlight the extreme values—for example, use bold for the highest and italics for the lowest—and include a note at the bottom of the table to explain the formatting. This would be useful for Tables 3 and 4. Even though Figures 2 and 3 display the lowest and highest values, referring back to them while reading the tables (which appear on a different page) is not straightforward.

**Results of the Regression Analysis**

Page , lines 252-255, you talk about unity and variety. They are similar to typicality and novelty, but they are different product aesthetic properties. So, is this a mistake? Please revise. See UMA project to understand that unity and variety are different.

**Discussion**

For understanding results related to typicality, I recommend this more recent reference. It could help you justify why certain typicality measures have higher SD than other measures (of typicality in other categories or novelty itself). As well as differences between category types (open vs. closed).

Ceballos, L. M., Hodges, N., & Watchravesringkan, K. (2021). Decoding typicality in apparel products: an investigation of consumer perceptions. *International Journal of Fashion Design, Technology and Education*, *14*(1), 37-47.

**General notes on the paper**

For easier understanding, please revise and always present first definitions, results, and so on, of typicality and then, novelty. For instance, you start with typicality first, and novelty second. And then, in Introduction, page 2/16, lines 47-52, you define novelty first, and then typicality. The same order and consistency helps with fluency in reading. The same logic should be applied to open vs. closed categories, and lighting and ceramic vases. Please use the same order throughout the whole paper. Notice that in page 3/16 lines 86-91, you mention lightings first, and vases second. But in result section you usually present vases first, and lightings second. The same for materials and methods, there are diferent orders between explanation and Fig. 1, for example.

**Discussion**

When you state theory that confirms your findings, you should always cite references, such as a when you mention processing fluency or how you explain how novel designs offer more stimulation. Remember that the goal is to explicitely connect to previous literature to extender statistical findings. The whole discussion only cites 2 references…

**Conclusion**

Something that is implicit, but relevant, and should be made explicit, is that your study also confirms the MAYA principle for both categories, as the both factors do interact and explain aesthetic preference in your results.
