# Supplementary material for: Practical research on the boundaries of MAYA design principles with ceramic products as the carrier
Source: PLoS One. 2026 Apr 2;21(4):e0342855. doi: 10.1371/journal.pone.0342855 (PMC13046151; doi:10.1371/journal.pone.0342855)
Supplement: Attachment — Submitted filename: Response to Reviewers.docx [file pone.0342855.s005.docx]

Contents

[Editor and Reviewer 1	1](#_Toc1948692164)

[Reviewer 2	10](#_Toc396099951)

Dear editor,

Thanks for your letter and for reviewers’s comments concerning our manuscript entitled “Practical research on the boundaries of MAYA design Principles with Ceramic”(Manuscript ID:PONE-D-25-32602). Those comments are all valuable and helpful for revising and improving our paper. We have studied all comments carefully and have made conscientious correction. Revised portion are marked in the paper. The responds to the reviewers’s comments are as flowing:

# Editor and Reviewer 1

**Comment 1:Clarify writing and improve consistency:** Revise unclear sentences (e.g., in the abstract) and maintain consistent order when presenting key concepts like typicality vs. novelty and vases vs. lighting.

**Response:** Four unclear sentences were identified and revised: one in the Abstract, four in the Introduction, and one in the Results section, as detailed in **Table 1**.The order of key concepts—poor vs rich categories typicality vs. novelty and ceramic vases vs. Ceramic lighting items—was made consistent throughout the manuscript. Revisions were made in the Abstract , Introduction , Materials and Methods , Results , and Conclusion and mark with blue font.

Table 1. Revisions of unclear sentences

| Location | Revised text |
| --- | --- |
| Abstract (typicality,  novelty) |  |
| Introduction (1)  (aesthetic \aesthetic preferences) |  |
| Introduction (2)  (typicality,  novelty) |  |
| Introduction (3)  (CM model) |  |
| Introduction (4)  (stumuli) |  |
| Results  ((typicality,  novelty) |  |

**Comment 2: Strengthen methodological transparency:** Add details about the expert panel, stimulus selection, and measurement procedures, including justifications for construct-scales choices and sources.

**Response：**We appreciate the reviewer’s suggestion. To enhance methodological transparency, we have added detailed descriptions of the expert panel, stimulus selection, and measurement procedures. Specifically, we now include:

**一、Expert panel:** The expert panel consisted of five members from the Unified Model of Aesthetics (UMA) research team (page 4, lines 135–136).

**二、Stimulus selection:** The process and rationale for selecting the stimuli (ceramic vases and ceramic lighting items) have been elaborated in the same section (page 4, lines 131–141).The detailed selection process is shown in Figure 1.

Figure 1:Stimulus Selection Process

**三、Measurement procedures:** In the current study, consistent with prior research on cognitive-level aesthetic preferences, we employed a 7-point Likert scale for all evaluations. The constructs of typicality, novelty, and aesthetic preference (aesthetic pleasure) were operationalized using items adapted from Blijlevens et al.^[1]^, ensuring comparability with established measures^[2],^^[3],^^[4],^^[5],^^[6]^.

The use of single-item measures is theoretically justified and consistent with previous work^2,3,4,5^. Specifically, we followed Rossiter’s C-OAR-SE procedure, which supports single-item measurement for concrete and easily understood constructs^[7]^. Moreover, as Bergkvist and Rossiter demonstrated, for “doubly concrete” constructs—where both the object and attribute are concrete—single-item measures perform comparably to multi-item measures in terms of predictive validity^[8]^.

The measurement procedure and the evaluation process are illustrated in Figure 2. These clarifications have been incorporated into page 6， lines 181–202 of the revised manuscript.

Figure 2：Measurement procedure

**Comment 3: Enhance rigor and scientific contribution:** Include formal hypotheses, verify construct definitions and usage (e.g., unity or typicality? Aesthetic pleasure or aesthetic preference?), and support theoretical claims in the discussion with relevant citations.

**Response：**We appreciate the reviewer’s insightful suggestions. In response, we have taken the following steps to enhance the rigor and scientific contribution of our study:

1. **Formal Hypotheses:** Previous research has shown that closed categories tend to favor high typicality and low novelty^1，2，3，4^, whereas open categories tend to favor low typicality and high novelty^4^. However, this pattern has not yet been tested in the context of ceramic products. Therefore, the present study proposes the following hypothesis: For ceramic products, open categories are more tolerant of novelty than closed categories (page 4, lines 117–122).

In the discussion section, we explicitly note that the results of the present study support this hypothesis. Specifically, the findings indicate that open categories of ceramic products are indeed more tolerant of novelty than closed categories （page 12, lines 375–380).

**References**

1. Yahaya M. Investigating typicality and novelty through visual and tactile stimuli (Doctoral dissertation, Swinburne)
2. Ma J, Yahaya MF, Tai L, Whitfield A, Barron D. Exploring Smartwatch Aesthetic Preferences Through the Unified Model of Aesthetics. Empirical Studies of the Arts. 2025 Jan 9:02762374241309880.
3. Ding S, Yahaya MF, Abdul Rahman AR. Examining the multidimensional impact on soft drink packaging preferences through the unified model of aesthetics. Scientific Reports. 2025 Feb 8;15(1):4782.
4. Chen, Shaokang, et al. ”Categorization and Aesthetic Preference: Examining Typicality and Novelty Across Rich and Poor Categories.” Empirical Studies ofthe Arts (2025): 02762374251371282.

**二、Construct Definitions and Usage:** While the focus of this study is on aesthetic preference, we adopted the Aesthetic Pleasure in Design (APiD) scale rather than traditional aesthetic preference (AP) scales for the following reasons:

1) **Definition and theoretical basis:** Aesthetic pleasure is defined as the immediate pleasurable response derived from directly perceiving an object, independent of its functional or utilitarian value ^1^. As such, it constitutes the affective foundation of aesthetic preference. Measuring aesthetic pleasure therefore effectively captures the core of aesthetic preference in a perceptual context.
2) **Reliability and validity:** The APiD scale is validated and widely applied in subsequent research ^2，3，4，5^, whereas existing AP scales are heterogeneous and lack consensus, with multiple versions differing in content and measurement approach.
3) **Comparability:** By using APiD items, we ensure that our measures are reliable, valid, and comparable to prior studies, avoiding the inconsistencies inherent in AP scales.

For these reasons, the APiD scale was deemed the most appropriate instrument to operationalize aesthetic experience in this study.The detailed content is shown in the following figure.

**References**

1. Blijlevens J, Thurgood C, Hekkert P, Chen LL, Leder H, Whitfield TW. The Aesthetic Pleasure in Design Scale: The development of a scale to measure aesthetic pleasure for designed artifacts. Psychology of Aesthetics, Creativity, and the Arts. 2017 Feb;11(1):86.
2. Yahaya M. Investigating typicality and novelty through visual and tactile stimuli (Doctoral dissertation, Swinburne)
3. Ma J, Yahaya MF, Tai L, Whitfield A, Barron D. Exploring Smartwatch Aesthetic Preferences Through the Unified Model of Aesthetics. Empirical Studies of the Arts. 2025 Jan 9:02762374241309880.
4. Ding S, Yahaya MF, Abdul Rahman AR. Examining the multidimensional impact on soft drink packaging preferences through the unified model of aesthetics. Scientific Reports. 2025 Feb 8;15(1):4782.

5. Chen, Shaokang, et al. ”Categorization and Aesthetic Preference: Examining Typicality and Novelty Across Rich and Poor Categories.” Empirical Studies ofthe Arts (2025): 02762374251371282.

**Comment 4:Improve presentation of results and key findings:** Make figures and tables easier to interpret by adding labels, totals, and clarifications; visually highlight key values. Explicitly state in the conclusion that the study confirms the MAYA principle across both product categories.

**Response：**We appreciate the reviewer’s suggestions regarding the presentation of results and key findings. In response, we have made the following improvements:

**一、Figures 2 and 3,Tables 3 and 4：**

Thank you for your helpful suggestion. In the revised manuscript, Figure 2 has been integrated with Table 3, and Figure 3 has been integrated with Table 4 to present the corresponding data and visual stimuli together. This combination allows for easier comparison between numerical results and the visual representations of the ceramic vases and lighting items. To further improve readability, miniature stimulus images have been placed below each data point, with the highest values highlighted in bold, the lowest in italics, and a light gray background applied for visual clarity.

**二、Conclusion:** We have explicitly stated in the conclusion that the study confirms the MAYA principle (Most Advanced Yet Acceptable) across both product categories examined (ceramic vases and ceramic lighting items), linking the empirical results directly to the theoretical framework （page 12, lines 388–394)

# Reviewer 2

**Comment 1:**Concept

On p. 3, line 70, the authors invoke Whitfield’s (2005) model (named Categorical-Motivational model, the CM model), which states that object category influences the weight of typicality and novelty in shaping aesthetic preference.

As I understand, the CM model includes some types of categories, with open and closed categories at two extremes, partially open categories between these two extremes, and rich and poor categories within the partially open categories (see some early work from Whitfield, 1983, 2000, 2005, 2009; Whitfield & Slatter, 1979; and some empirical evidence, e.g., Suhaimi, 2023; Chen et al., 2025). There is something unclear about why the authors defined vases are open categories and lightings are closed categories. Thus, I would ask the authors to clarify the reasons. This may help the reader to understand better.

**Response：**We sincerely thank the reviewer for this insightful comment. In the revised manuscript, we have clarified the definition of the CM model (see [page 3, lines 77–79]) and further explained the relationships among closed, partially open, and open categories, as well as their connection to poor and rich categories (see [page 3，lines 79–100]). In addition, we have specified that ceramic vases are classified as poor categories, whereas ceramic lightings are classified as rich categories (see [page 4，lines 111–114]).

Comment 2:Stimulus-selection procedure
As the study aims to compare the typicality and novelty in shaping aesthetic preference across two types of products. It is necessary to select appropriate stimuli to control the independent variables, typicality and novelty, at a comparable level. In more direct words, you cannot select ten typical products versus ten novel products. I would ask the authors to clarify the procedure for how the two types of products are at a comparable level in typicality and novelty.

**Response：We sincerely thank the reviewer for this important comment concerning the comparability of typicality and novelty across the two product types. In the revised manuscript, we have added a detailed explanation of the stimulus-selection procedure to clarify how the stimuli were selected and controlled** (page 4, lines 131–141)**.**

**Comment 3:**Positive correlation between typicality and novelty

**Response：**We thank the reviewer for raising this insightful point. In the revised manuscript, we have provided a clearer discussion of the relationship between typicality and novelty (page 11， lines 319–329).

**Comment 4:Figures 2 and 3,Tables 3 and 4**

Thank you for your helpful suggestion. In the revised manuscript, Figure 2 has been integrated with Table 3, and Figure 3 has been integrated with Table 4 to present the corresponding data and visual stimuli together. This combination allows for easier comparison between numerical results and the visual representations of the ceramic vases and lighting items. To further improve readability, miniature stimulus images have been placed below each data point, with the highest values highlighted in bold, the lowest in italics, and a light gray background applied for visual clarity.

**Comment 5:**Tables 7, 8, 9 & 10: Combine them into a single table that directly shows the correlation result.

**Response：**Thank you for your constructive suggestion. In the revised manuscript, Tables 7–10 have been merged into a single comprehensive table that presents all correlation results in an integrated format. This modification allows for easier comparison across variables and categories, reducing redundancy and improving the clarity of data presentation. The updated table is now presented as Table 5 (see page 9 of the revised manuscript).

**Comment 6:**Report the demographic and reliability results before data analysis.

**Response：**We sincerely thank the reviewer for this valuable suggestion. In the revised manuscript, we have reorganized the Methods and Results sections to present participant demographics and, regarding reliability, relevant information prior to the main statistical analyses. Specifically:

**一、Demographics:** We provide detailed information on participants’ age, gender, educational background, and relevant experience with the product categories. This allows readers to better understand the sample characteristics and the generalizability of the findings (page 6,7， lines 224–234).

1. Regarding reliability, we used **single-item measures** for the main variables (e.g., aesthetic preference,typicality,novelty), and therefore traditional reliability indices such as Cronbach’s alpha are not applicable. We have clarified this point in the revised manuscript to avoid any potential confusion (page 6, lines 191–202).

1. Blijlevens J, Thurgood C, Hekkert P, Chen LL, Leder H, Whitfield TW. The Aesthetic Pleasure in Design Scale: The development of a scale to measure aesthetic pleasure for designed artifacts. Psychology of Aesthetics, Creativity, and the Arts. 2017 Feb;11(1):86.
2. Suhaimi SN, Kuys B, Barron D, Li N, Rahman Z, Whitfield A. Probing the extremes of aesthetics: The role of typicality and novelty in the aesthetic preference of industrial boilers. Empirical Studies of the Arts. 2023 Jan;41(1):216-30.
3. Yahaya M. Investigating typicality and novelty through visual and tactile stimuli (Doctoral dissertation, Swinburne)
4. Ma J, Yahaya MF, Tai L, Whitfield A, Barron D. Exploring Smartwatch Aesthetic Preferences Through the Unified Model of Aesthetics. Empirical Studies of the Arts. 2025 Jan 9:02762374241309880.
5. Ding S, Yahaya MF, Abdul Rahman AR. Examining the multidimensional impact on soft drink packaging preferences through the unified model of aesthetics. Scientific Reports. 2025 Feb 8;15(1):4782.
6. Chen, Shaokang, et al. ”Categorization and Aesthetic Preference: Examining Typicality and Novelty Across Rich and Poor Categories.” Empirical Studies ofthe Arts (2025): 02762374251371282.
7. Rossiter, John R. ”The C-OAR-SE procedure for scale development in marketing.” International journal of research in marketing 19.4 (2002): 305-335.
8. Bergkvist, Lars, and John R. Rossiter. ”The predictive validity of multiple-item versus single-item measures of the same constructs.” Journal of marketing research 44.2 (2007): 175-184. Bergkvist, Lars, and John R. Rossiter. ”The predictive validity of multiple-item versus single-item measures of the same constructs.” Journal of marketing research 44.2 (2007): 175-184.
