# Supplementary material for: Practical research on the boundaries of MAYA design principles with ceramic products as the carrier
Source: PLoS One. 2026 Apr 2;21(4):e0342855. doi: 10.1371/journal.pone.0342855 (PMC13046151; doi:10.1371/journal.pone.0342855)
Supplement: Attachment — Submitted filename: Response_to_Reviewers_auresp_2.docx [file pone.0342855.s006.docx]

**Contents**

[Reviewer 1	1](#_Toc1948692164)

Dear editor,

Thanks for your letter and for reviewers’s comments concerning our manuscript entitled “Practical research on the boundaries of MAYA design Principles with Ceramic”(Manuscript ID:PONE-D-25-32602). Those comments are all valuable and helpful for revising and improving our paper. We have studied all comments carefully and have made conscientious correction. Revised portion are marked in the paper. The responds to the reviewers’s comments are as flowing:

# Reviewer 1

**Comment 1: I note that the manuscript formulates a single hypothesis. While this is not common, it is acceptable for PLOS ONE, given the journal’s emphasis on methodological rigor rather than the number of hypotheses. However, the current presentation does not sufficiently emphasize this hypothesis as the central organizing element of the study.
With minor revisions, I recommend making the hypothesis more visible and clearly articulated throughout the manuscript. The Conclusions should then explicitly state whether the hypothesis is supported or not, as this section should tie together the study’s objectives, analyses, and main contributions. While a similar point is addressed in the final part of the Discussion, I believe it should be reiterated in the Conclusions to clearly close the loop of the hypothesis testing.**

**Response:** Thank you for this valuable suggestion. Following your advice, we have revised the manuscript to more clearly emphasize the proposed hypothesis as the central organizing element of the study. Specifically:

In the Introduction, we explicitly identify the hypothesis as the core guiding principle of the research and clarify that all subsequent analyses are designed to test this hypothesis.

In the Discussion, we explicitly link the empirical findings back to the hypothesis and state that the results provide empirical support for it.

In the Conclusions, we reiterate whether the hypothesis is supported and clearly complete the loop of hypothesis testing by summarizing how the study’s objectives, analyses, and main contributions align with the proposed hypothesis.

These revisions ensure that the hypothesis is consistently highlighted throughout the manuscript and that its empirical support is clearly stated in the Conclusions, as recommended. All revisions are detailed in **Table 1**.

Table 1. Details of Hypothesis-related Revisions

| Location | Revised text |
| --- | --- |
| Introduction  （page 4, lines 120–127） |  |
| Discussion  （page 12, lines 380–383） |  |
| Conclusion  （page 12, lines 394–397） |  |
| Conclusion  （page 13, lines 411–416） |  |

**Comment 2: Tables and Figures 2 and 3 are now fantastic and easier to understand! Great way to unify and make it comparable and contrasting. When explaining figures in which the highest and lowest values are highlighted or italicized, please include a note explaining these markings, either in the text or, ideally, in a note below each specific figure. These markings may not be obvious to a reader.**

**Response：**We appreciate the reviewer’s positive feedback and helpful suggestion. In response, we have added explicit explanatory notes to Figures 2 and 3, clarifying that bold values indicate the highest mean scores and italicized values indicate the lowest mean scores. In addition, we have included corresponding explanations in the main text when the figures are first introduced. These revisions improve the clarity and interpretability of the figures for readers.

1.**Main text**
2.**Figures 2 and 3**
